# Mechanisms of Exercise Training in Ameliorating Cognitive Function in Patients With Depression: A Narrative Review of Molecular Targets and Clinical Prescriptions

**DOI:** 10.1002/ejsc.70272

**Published:** 2026-10-12

**Authors:** Zhaohui Guo, Lingling Dong, Jessie J. X. Lin, Yanping Duan, Weizhi Liu, Wanting Jiang, Cong Liu, Shuqi Jia, Yagang Song, Feng Ding, Zhenwen Xie, Mingyue Yin, Kang Zhu, Xiaochen Ma, Peng Wang, Chen Wei, Felipe Barreto Schuch, Xing Wang

**Affiliations:** ^1^ School of Physical Education Shanghai University of Sport Shanghai China; ^2^ Department of Psychiatry Shanghai Baoshan District Mental Health Center Shanghai China; ^3^ Department of Rehabilitation Sciences The Hong Kong Polytechnic University Hong Kong China; ^4^ Department of Sports and Health Sciences Hong Kong Baptist University Hong Kong China; ^5^ Department of Basic Psychology Naval Medical University Shanghai China; ^6^ School of Athletic Performance Shanghai University of Sport Shanghai China; ^7^ School of Coaching Shanghai University of Sport Shanghai China; ^8^ School of Sports Science Shenyang Normal University Shenyang China; ^9^ Institute for Mental and Physical Health and Clinical Translation Deakin University Geelong Australia; ^10^ Institute for Physical Activity and Nutrition Deakin University Melbourne Australia; ^11^ Faculty of Health Sciences Universidad Autónoma de Chile Providencia Chile

**Keywords:** cognitive function, depression, exercise training, mechanisms

## Abstract

Depression is frequently accompanied by core cognitive impairments, notably in executive function and memory. Although the ameliorative effects of exercise training have been widely corroborated, the evidence regarding its underlying mechanisms remains fragmented. To provide a comprehensive and integrated view, this review proposes a testable, cross‐scale “peripheral‐central‐behavioral” hypothetical mechanistic model to explain the effects of exercise on cognition. The core theoretical contribution of this study lies in explicitly postulating a “molecule‐structure‐function” cross‐level mediation hypothesis. The exercise‐induced upregulation of peripheral neurotrophic factors must be mediated by a core “structural mediator”, specifically the enhanced white matter integrity of key tracts, to ultimately drive the “electrophysiological output” of optimized cerebral information processing efficiency. Furthermore, this model proposes the temporal cascade effects of neuro‐psychological interactive regulation, distinguishing between early subacute HPA axis normalization and mid‐to‐long‐term reward system activation. Crucially, recognizing the profound translational gap between biological mechanisms and clinical application, this review emphasizes that optimal exercise prescriptions cannot be dictated solely by physiological targets. Addressing the heterogeneity of depressed individuals, we propose a precision intervention framework that meticulously balances neurobiological efficacy by tailoring specific modalities and optimal dose‐response thresholds to distinct depression subtypes. Ultimately, this review provides a theoretical framework for future stratified randomized controlled trials (RCTs) based on longitudinal multimodal co‐measurements, paving the way for clinically viable, predictable, and highly individualized exercise prescriptions.

## Highlights

- Novel Mechanistic Framework: This review proposes a cross‐scale “peripheral‐central‐behavioral” hypothetical mechanistic model to explain exercise‐induced cognitive recovery in depression. It postulates a “molecule‐structure‐function” mediation where peripheral neurotrophic factors enhance central white matter integrity to optimize electrophysiological output.
- Temporal Cascade Effect: The framework hypothesizes a potential temporal sequence of neuro‐psychological interactive regulation. This predictive timeline distinguishes between early subacute HPA axis normalization to mitigate neurotoxicity and mid‐to‐long‐term reward system activation to drive executive function recovery.
- Systemic Molecular Crosstalk: It integrates advanced peripheral‐central crosstalk mechanisms, including FGF21 signaling, gut microbiota‐derived short‐chain fatty acids, and alternative energy metabolism remodeling, expanding the focus from isolated pathways to a systemic multi‐omics network.
- Precision Clinical Prescriptions: Addressing individual heterogeneity, the study proposes transitioning from generic step counts to a precision intervention framework. It tailors specific exercise modalities and precise physiological intensities to distinct depression subtypes for optimal neurobiological efficacy.

**Abbreviations**
ACC: Anterior Cingulate Cortex
AMPK: AMP‐Activated Protein Kinase
ANLS: Astrocyte‐Neuron Lactate Shuttle
BBB: Blood‐Brain Barrier
BDNF: Brain‐Derived Neurotrophic Factor
BHB: *β*‐Hydroxybutyrate
CEN: Central Executive Network
CT: Concurrent Training
DA: Dopamine
DLPFC: Dorsolateral Prefrontal Cortex
DMN: Default Mode Network
DTI: Diffusion Tensor Imaging
EEG: Electroencephalogram/Electroencephalography
ERPs: Event‐Related Potentials
FA: Fractional Anisotropy
FAA: Frontal Alpha Asymmetry
FGF21: Fibroblast Growth Factor 21
fMRI: Functional Magnetic Resonance Imaging
HIIT: High‐Intensity Interval Training
HPA: Hypothalamic‐Pituitary‐Adrenal
IGF‐1: Insulin‐like Growth Factor‐1
IL‐10: Interleukin‐10
IL‐1*β*
: Interleukin‐1 beta
IL‐6: Interleukin‐6
LTP: Long‐Term Potentiation
MBE: Mind‐Body Exercise
MDD: Major Depressive Disorder
MQC: Mitochondrial Quality Control
MRI: Magnetic Resonance Imaging
MS‐MHb: Medial Septum‐Medial Habenula
mTOR: Mammalian Target of Rapamycin
NRF1: Nuclear Respiratory Factor 1
OFC: Orbitofrontal Cortex
OPCs: Oligodendrocyte Precursor Cells
PFC: Prefrontal Cortex
PVN‐NAc: Paraventricular Nucleus of the hypothalamus to Nucleus Accumbens
RCTs: Randomized Controlled Trials
ROS: Reactive Oxygen Species
RT: Resistance Training
SCFAs: Short‐Chain Fatty Acids
SNR: Signal‐to‐Noise Ratio
TCA: Tricarboxylic Acid
TFAM: Mitochondrial Transcription Factor A
TNF‐*α*
: Tumor Necrosis Factor‐alpha
5‐HT: Serotonin

## 1 Introduction

Depression has emerged as the foremost global mental health challenge, accounting for 10.3% of all psychiatric disorders (Herrman et al. 2022). Data from the Global Burden of Disease study (1990–2017) indicated that the total number of individuals suffering from depression worldwide had exceeded 350 million, representing a 49.86% increase over this period (Liu et al. 2020). As a pervasive mental health disorder, depression exerts profound negative impacts, severely disrupting patients' daily lives and social functioning (Jia et al. 2024).

Depression clinical manifestations extend beyond mere depressed mood to encompass significant cognitive impairments, which compromise core domains including attention, executive function, memory, and information processing speed (Kinney‐Huang et al. 2023; Nooij et al. 2020). Studies have demonstrated that when executing complex tasks, depressed patients frequently exhibit prolonged reaction times and reduced accuracy, particularly in scenarios demanding highly sustained attention and rapid decision‐making (Pan et al. 2025; Huang et al. 2023). Crucially, these cognitive deficits are intrinsically linked to the severity of the depression, the risk of relapse, and the treatment response (Kriesche et al. 2023). Multiple studies indicate a positive correlation between cognitive decline and symptom severity; namely, the more severe the depressive symptoms, the greater the degree of cognitive impairment (Dai et al. 2024). Furthermore, cognitive dysfunction may portend the risk of recurrent episodes, as evidenced by persistent cognitive decline even after the remission of depressive symptoms (M. H. Huang, Chan, et al. 2022). This underscores that cognitive assessment must constitute an integral component of the therapeutic process to facilitate the early detection of potential relapse risks. Consequently, the early identification of and intervention for cognitive impairment holds significant implications for improving patient prognoses. Strategies to prevent and mitigate cognitive decline in patients with depression have become a focal point in both academic and clinical research.

The cognitive deficits observed in depressed patients are closely intertwined with multiple neural mechanisms, notably structural brain abnormalities, neural network dysfunction, and alterations in inflammatory responses and neurotrophic factors (Tian et al. 2025; Jellinger 2023; Zacková et al. 2021; Hassamal 2023). First, structural brain anomalies are particularly pronounced in MDD. Numerous studies highlight a pervasive volume reduction in critical brain regions, including the prefrontal cortex (PFC), hippocampus, and anterior cingulate cortex (ACC) (Zhao et al. 2014; Xu et al. 2024). For instance, impairments in executive function and emotion regulation are associated with aberrant functional activity in the PFC specifically, the dorsolateral prefrontal cortex (DLPFC) (Grieve et al. 2013), which is deeply involved in cognitive processes such as working memory and inhibitory control. Moreover, diminished hippocampal volume is strongly correlated with memory deficits (Kaiser et al. 2015), and depressed patients exhibit significantly lower hippocampal activation during memory tasks compared with healthy controls. As a pivotal intersection for emotional and cognitive processing, structural and functional alterations in the ACC may further exacerbate depressive symptoms and cognitive deficits (Grieve et al. 2013). Second, neural network dysfunction may act as a potential mechanism. Current neuroimaging evidence reveals significant topological abnormalities in the functional brain networks of patients with MDD. Not only is there hyperactivation within the default mode network (DMN), but more critically, the functional connectivity between the central executive network (CEN) and emotion regulation circuits (e.g., the limbic system) is significantly reduced (Kaiser et al. 2015). This network “decoupling” phenomenon weakens the prefrontal cortex's top‐down inhibitory capacity on negative emotions, thereby compromising cognitive control (Clasen et al. 2014). At the neuroelectrophysiological level, the electroencephalogram (EEG) spectral characteristics of depressed patients reflect abnormal cortical arousal. This is manifested as abnormally elevated power spectral density in low‐frequency bands (e.g., Theta and Alpha waves) during the resting state, typically associated with cortical inhibition and sluggish information processing. Conversely, high‐frequency band activity (e.g., Gamma waves), which is closely linked to cognitive integration and attention, is significantly diminished (Newson and Thiagarajan 2018). Additionally, frontal alpha asymmetry (FAA) specifically, relatively increased alpha activity in the left frontal lobe, indicating left‐hemisphere hypoactivation has been validated as a biomarker for impaired emotion regulation and approach‐motivation deficits in depression (Thibodeau et al. 2006; Li et al. 2026). Concurrently, dysregulation of neurotransmitter systems provides a molecular explanation for cognitive impairment. The decreased transmission efficiency of serotonin (5‐HT) and dopamine (DA) is not only the core etiology of depressed mood but also directly impairs synaptic plasticity in the PFC (Hammar and Ardal 2009). The hypoactive state of dopaminergic pathways is directly linked to deficits in encoding Reward Prediction Errors, leading to significant functional hypoactivation in core reward regions such as the orbitofrontal cortex (OFC), ventral striatum, and PFC (Dunlop and Nemeroff 2007). This blunting of the “reward circuit” (anhedonia) not only reduces pleasure but also severely interferes with reward‐based learning, decision‐making, and executive functions (Pizzagalli 2014; Zhang et al. 2013).

Beyond macroscopic networks and electrophysiological alterations, the interplay between neuroimmunity and neurotrophic factors is vital. Extensive research has demonstrated significantly reduced serum levels of Brain‐Derived Neurotrophic Factor (BDNF) in patients with MDD (Cavaleri et al. 2023). This molecular deficit directly undermines synaptic plasticity and Long‐Term Potentiation (LTP) in the hippocampus and PFC, thereby impeding neurogenesis and memory consolidation (Molendijk et al. 2014; Zhang et al. 2016). Simultaneously, the overactivation of the immune system triggers a cascade of neuroinflammatory responses. MDD patients often exhibit significantly elevated pro‐inflammatory cytokines, notably TNF‐α, IL‐1β, and IL‐6 (Dowlati et al. 2010). These peripheral inflammatory factors can cross the blood‐brain barrier, inducing abnormal microglial activation, generating neurotoxicity, and interfering with neurotransmitter synthesis (Miller and Raison 2016). Crucially, a chronic inflammatory environment further suppresses BDNF expression, forming a vicious “inflammation‐neurotrophic deficit” cycle. This dual molecular strike significantly exacerbates executive dysfunction and information processing delays, forming an important biological basis for the stubbornness of cognitive impairment in depression (Rosenblat et al. 2015; Calabrese et al. 2014).

In summary, the neural mechanisms underlying cognitive impairment in depression are multifaceted, involving structural alterations, functional dysregulation, and neurochemical/inflammatory abnormalities. Elucidating these mechanisms not only deepens our understanding of the pathophysiology of depression but also provides a crucial basis for developing targeted interventions.

Against this backdrop, Exercise therapy, as a multi‐target non‐pharmacological intervention, demonstrates unique advantages (Singh et al. 2023; Vancampfort et al. 2025; Noetel et al. 2024; Brobakken et al. 2022). Accumulating research suggests that exercise training can modulate neurobiological mechanisms, such as enhancing brain functional connectivity and improving psychological state impairment, thereby positively impacting depression‐related cognitive impairment (C. Liu, Chen, et al. 2026; Zhao et al. 2020; Ren et al. 2024). A systematic review and meta‐analysis (Dauwan et al. 2021) showed that exercise training significantly improves mood in depressed patients with a positive dose‐response relationship, alongside significant cognitive improvements. Furthermore, comparative studies have found that the clinical efficacy of exercise in ameliorating psychiatric disorders has effect sizes comparable to those found in pharmacotherapy and other interventions (Noetel et al. 2024; Heissel et al. 2023). However, the mechanisms by which exercise improves cognitive function in depression have yet to be systematically organized and elucidated.

In recent years, with the rapid advancement of multimodal neuroscience technologies, integrating evidence from neuroimaging, peripheral biomarkers, and neurophysiology has become a critical window into this mechanism. Functional magnetic resonance imaging (fMRI) studies reveal that long‐term, regular exercise can reverse hippocampal atrophy and enhance functional connectivity between the PFC and the limbic system, closely correlating with the recovery of memory and executive functions (Firth et al. 2018; Schmaal et al. 2017). Peripheral biomarker evidence indicates that exercise can upregulate BDNF expression and reduce pro‐inflammatory cytokine concentrations (e.g., IL‐6), thereby enhancing neuroplasticity and mitigating the detrimental effects of neuroinflammation on cognition (Szuhany et al. 2015; Hamer et al. 2009; Guo et al. 2024). Electrophysiological evidence demonstrates that exercise interventions effectively modulate EEG activity in depressed patients (Li et al. 2024). Cross‐sectional findings show that frontal EEG indices including beta2 power at (FP1, FP2) and delta power at (F3, F4) and (F7, F8) are negatively correlated with depression scores and verbal working memory reaction times, and positively correlated with total physical activity scores (Ren et al. 2025). Randomized controlled trials provide evidence that exercise significantly increases the N2 amplitude and shortens the latency of event‐related potentials (ERPs), thereby reflecting the optimization of cerebral information processing speed and attentional resources (Yun et al. 2025).

In summary, while extensive research has corroborated the cognitive benefits of exercise for depression, current mechanistic investigations often remain confined to isolated biological levels, predominantly stagnating at the static, parallel enumeration of evidence. Consequently, there is a profound dearth of theoretical models that deeply integrate molecular mechanisms with brain network remodeling across hierarchical levels. To bridge this critical research gap, this review not only systematically synthesizes recent evidence but also proposes a novel, falsifiable, cross‐scale “peripheral‐central‐behavioral” hypothetical mechanistic model. We explicitly articulate specific “molecule‐structure‐function” mediation hypotheses and profoundly elucidate the potential temporal cascade of exercise interventions, as well as specific predictive pathways tailored to distinct depression subtypes. Crucially, we recognize that the current evidence evaluating exercise‐induced changes in neurobiological markers among depressed individuals remains limited and inconsistent, with numerous trials and meta‐analyses exhibiting methodological limitations and null findings. Therefore, rather than proposing immediate clinical guidelines, we position this framework as a promising conceptual model awaiting rigorous empirical testing. While this review delineates potential mechanisms by which exercise remodels cognitive control networks, we strongly advocate for well‐designed longitudinal studies to validate these hypotheses. Ultimately, only when substantiated by robust and reproducible evidence can such a model translate into clear clinical recommendations and advance the development of precision exercise prescriptions guided by objective biomarkers.

## 2 Literature Search Strategy and Methodological

To maximize scientific transparency and methodological reproducibility, a comprehensive literature search was executed across core electronic databases, including PubMed, Web of Science, and Scopus, capturing literature from their respective inceptions up to April 2026. The search strategy utilized Medical Subject Headings (MeSH) and free‐text terms, connected via Boolean operators. The primary search syntax encompassed combinations of the following terms: (“Depression” OR “Major Depressive Disorder” OR “MDD”) AND (“Exercise” OR “Physical Activity” OR “Exercise Training” OR “Aerobic” OR “Resistance” OR “Concurrent Training” OR “High‐Intensity Interval Training” OR “HIIT” OR “Mind‐Body”) AND (“Cognition” OR “Cognitive Function” OR “Memory” OR “Executive Function” OR “Neuroplasticity”) AND (“Mechanism” OR “Neurobiology” OR “Biomarkers” OR “Molecular” OR “Gut Microbiota” OR “Short‐Chain Fatty Acids”). To ensure a rigorous scope, the search was predominantly restricted to peer‐reviewed articles published in English, and backward citation tracking (snowballing) was manually performed on the reference lists of pivotal studies to identify any additionally relevant mechanistic literature.

Despite this rigorous retrieval process, we must objectively acknowledge the inherent methodological limitations of a narrative review framework. Unlike compliant systematic reviews or quantitative meta‐analyses, this synthesis did not employ standardized risk of bias appraisal tools. Consequently, the narrative synthesis is inherently susceptible to potential literature selection bias, and the integration of highly heterogeneous multi‐scale evidence, spanning from murine models to human clinical trials, inevitably involves a degree of subjective interpretation by the authors. Therefore, rather than serving as an exhaustive or statistically definitive systematic appraisal, the integrated “peripheral‐central‐behavioral” mechanistic model proposed herein is explicitly designed as a robust, hypothesis generating conceptual scaffold. This framework urgently awaits rigorous empirical validation through future well‐powered biomarker‐guided longitudinal trials and focused on quantitative syntheses.

## 3 Effects of Different Exercise Modalities on Cognitive Function in Patients With Depression

### 3.1 Ameliorative Effects of Aerobic Exercise

When specifically examining populations with depression, aerobic exercise emerges as a highly efficacious intervention for cognitive rehabilitation. A recent comprehensive three‐level meta‐analysis specifically evaluated various exercise modalities and provided robust evidence that aerobic training, which comprised the majority of the analyzed clinical trials, significantly ameliorates the cognitive impairment strictly associated with depressive episodes (C. Liu, Chen, et al. 2026). To further substantiate these effects, a growing body of recent randomized controlled trials and domain‐specific meta‐analyses have illuminated the precise cognitive benefits of aerobic regimens in clinical settings. For instance, recent rigorously designed trials targeting inpatients with severe first‐episode depression demonstrated that moderate intensity aerobic exercise, when administered alongside standard therapy, significantly accelerates the recovery of comprehensive cognitive performance and processing speed compared to treatment as usual (C. Liu, Zhai, et al. 2026). Furthermore, updated evidence confirms that aerobic exercise yields particularly pronounced and targeted enhancements in specific subdomains, notably working memory, executive function, and verbal memory, thereby allowing depressed patients to better retain information and execute complex tasks (Ren et al. 2024; Zhu et al. 2026). These concrete empirical findings provide robust, population‐specific support, confirming that for depressed individuals, aerobic exercise acts as a precise clinical countermeasure to reverse disease‐related cognitive decline.

### 3.2 Ameliorative Effects of Resistance Training

The resistance training (RT) is rapidly emerging as a robust intervention specifically for alleviating depressive symptoms and associated cognitive deficits. A recent comprehensive meta‐analysis comprising 29 randomized controlled trials (Chang et al. 2025) has definitively established that RT yields clinically meaningful reductions in core depressive symptoms across diverse clinical phenotypes. Crucially, emerging empirical evidence in cognitive rehabilitation confirms the specific benefits of RT. Recent rigorously designed trials demonstrate that RT interventions significantly enhance global cognitive function in populations experiencing depressive symptoms. Specifically, these cognitive enhancements are consistently noted in domains such as visuospatial and executive functions, as well as sustained attention (Chen et al. 2023). Although the total volume of clinical trials examining the cognitive effects of RT in pure major depressive cohorts remains smaller than that of aerobic exercise, these recent high‐quality syntheses and trials highlight RT as a potent domain‐specific modality. Ultimately, current evidence supports RT as an effective clinical countermeasure capable of simultaneously improving both mood (O'Sullivan et al. 2023) and the distinct cognitive deficits associated with depression (Chang et al. 2025).

### 3.3 Ameliorative Effects of Mind‐Body Exercise

In addition to conventional training modalities, mind‐body exercise, encompassing practices such as Tai Chi, yoga, and Qigong, has garnered unprecedented clinical attention as a highly efficacious dual‐action therapy. Crucially, recent high‐quality evidence explicitly demonstrates that this modality can simultaneously ameliorate both core depressive symptoms and the specific cognitive impairments inherent to depression within the exact same clinical cohorts. A comprehensive Bayesian model‐based network meta‐analysis specifically evaluating adults diagnosed with major depressive disorder revealed that among various exercise types, mind‐body exercises such as Tai Chi and Qigong demonstrated the greatest cognitive benefits for this clinical population (Ge et al. 2025). To further corroborate these findings at the clinical trial level, a rigorously designed randomized controlled trial targeting the major depressive disorder population confirmed that a structured yoga intervention significantly reduced clinical depression severity on the Hamilton Rating Scale while concurrently yielding massive improvements in objective cognitive markers, including comprehensive cognitive assessment scores and neural processing speed (Sharma et al. 2025).

### 3.4 Ameliorative Effects of Concurrent Training

Concurrent training (CT), defined as the systematic integration of aerobic and resistance training, is frequently recommended due to its multi‐targeted theoretical rationale. However, foundational evidence elucidating the specific cognitive synergistic effects of CT is predominantly derived from aging populations (Northey et al. 2018; Colcombe and Kramer 2003). Classic meta‐analyses demonstrate that compared with single‐modality approaches, CT regimens exert a significantly more pronounced positive impact on global cognitive function (Northey et al. 2018; Colcombe and Kramer 2003; Fyfe et al. 2014). Furthermore, recent empirical evidence corroborates the psychological benefits of CT among populations experiencing psychological distress; for instance, a recent randomized controlled trial (RCT) indicated that CT significantly mitigates depressive and anxiety symptoms in physically inactive adults (Higham et al. 2025).

Nevertheless, translating this synergistic paradigm into the clinical treatment of depression reveals a notable empirical controversy. A recent comprehensive three‐level meta‐analysis explicitly evaluating exercise interventions in depressed cohorts highlighted a critical discrepancy: while single‐modality interventions (e.g., aerobic, resistance, and mind‐body exercises) demonstrated robust and significant efficacy in alleviating depressive symptoms, concurrent exercise regimens unexpectedly failed to yield statistically significant improvements (*p* > 0.05). Notably, however, the specific impact of CT on cognitive domains within this depressed cohort remained unanalyzed (C. Liu, Chen, et al. 2026).

Given that patients with depression typically exhibit pervasive cognitive deficits across domains such as memory and executive function, there is currently a conspicuous dearth of clinical validation regarding the use of CT regimens for this population. Consequently, future longitudinal studies must prioritize investigating whether the theoretical synergy of CT translates into tangible cognitive benefits for depressed patients, or whether underlying physiological interference blunts its overall clinical efficacy.

## 4 The Impact of Exercise “Dose‐Response” on Cognitive Function in Patients With Depression and Its Underlying Mechanisms

While traditional guidelines have suggested that interventions characterized by low to moderate intensity and a duration of at least 10 weeks are necessary for cognitive improvements (Bueno‐Antequera and Munguía‐Izquierdo 2020), recent high‐quality evidence highlights a more nuanced reality. Addressing this controversy, a comprehensive meta regression (Heissel et al. 2023) demonstrated that shorter exercise interventions, especially those utilizing moderate intensity and supervised formats, are actually associated with larger therapeutic effects for depressed cohorts. To establish the optimal prescription specifically for cognitive rehabilitation, a recent three‐level dose‐response meta‐analysis (C. Liu, Zhai, et al. 2026) provided critical threshold data. Their findings indicate that cognitive benefits become significantly more pronounced when moderate‐intensity exercise exceeds a cumulative volume of approximately 176 minutes per week. This underscores the imperative of individualizing exercise doses to meet this specific therapeutic threshold. Finally, addressing the underlying biological pathways, an adequate exercise volume does not merely promote generalized neuroplasticity; rather, reaching this optimal dose specifically triggers the upregulation of critical neurotrophins, such as brain‐derived neurotrophic factor, and facilitates targeted hippocampal structural remodeling (Souza et al. 2023; D'Angelantonio et al. 2022).

However, regarding the precise quantification of exercise dosage, a recent large‐scale dose‐response meta‐analysis (Ding et al. 2025; Pearce et al. 2022) reveals that the relationship between physical activity volume and depression risk is not strictly linear, but rather exhibits a specific threshold effect. Empirical evidence demonstrates that accumulating approximately 7000 steps per day serves as a critical inflection point for preventing the onset of depressive symptoms in general, non‐depressed populations. Compared to low baseline activity levels (e.g., 2000 steps), achieving this threshold reduces the risk of incident depression by approximately 22%, with the rate of additional preventive benefits plateauing beyond this juncture. While this discovery offers an accessible public health target, it is crucial to exercise strict caution when extrapolating these preventive findings directly to clinical treatment. Epistemologically and physiologically, a preventive threshold (e.g., 7000 steps) derived from healthy cohorts cannot be equated to a therapeutic dose required to rescue established cognitive deficits in patients with major depression. Furthermore, as a clinical metric, the “step count” exhibits significant limitations because it inherently lacks the capacity to quantify precise physiological intensity (e.g., percentage of maximum heart rate [%HRmax] or maximal oxygen uptake [%VO_2_max]). Since exercise‐induced neurobiological adaptations such as the targeted upregulation of BDNF, the reduction of neuroinflammation, and the optimization of neural network connectivity are intensity dependent (Szuhany et al. 2015; Cerqueira et al. 2019), relying solely on generic step volume is fundamentally insufficient. Therefore, future clinical guidelines for depressive cognitive rehabilitation must transition from volume based step counts to precise individualized physiological intensity prescriptions that guarantee the crossing of specific neurogenic thresholds.

Similarly, regarding the flexibility of exercise frequency, previous studies (Andersen et al. 2025), addressing the pervasive challenge of poor compliance, have demonstrated that concentrating physical activity into one or 2 days the “Weekend Warrior” pattern is equally efficacious as regular daily exercise in preventing incident depression, provided the total weekly volume is achieved. Although this accumulation model provides valuable flexibility for general physical activity promotion, its specific efficacy for cognitive rehabilitation in clinical depression cohorts remains to be rigorously confirmed.

On the other hand, exercise interventions do not necessarily adhere to a “more is better” paradigm. While moderate training exerts potent anti‐inflammatory and neuroprotective effects, excessively vigorous or exhaustive physical stress can paradoxically induce acute systemic inflammation and oxidative stress (Cerqueira et al. 2019). This physiological reality is particularly critical for clinical populations. A pivotal clinical investigation (M. H. Huang, Chen, et al. 2022) explicitly highlights that elevated inflammatory markers are directly associated with profound cognitive dysfunction and severe clinical exacerbations, including suicidal ideation, in patients with major depressive disorder. Consequently, prescribing excessively high exercise volumes or supramaximal intensities may inadvertently amplify the underlying neuroinflammatory cascades that drive cognitive deficits. This potential backfire effect further underscores the critical necessity of strictly adhering to the optimal, moderate intensity dose thresholds to ensure both cognitive benefits and psychiatric safety.

## 5 Integration of Multimodal Mechanisms: The Bio‐Psycho‐Behavioral Network of Exercise Training in Ameliorating Depressive Cognitive Impairment

### 5.1 Peripheral‐Central Crosstalk of Blood Biochemical Markers

Exercise primarily facilitates the recovery of cognitive function in depressed patients, potentially by altering the biochemical microenvironment within both the systemic circulation and the brain, thereby establishing a physical foundation for cognitive restoration.

First, aerobic exercise establishes a critical “nutritional supply line” between skeletal muscles and the brain. The brains of patients with depression typically exhibit a severe deficit in Brain‐Derived Neurotrophic Factor (BDNF). Exercise far transcends mere physical movement; the contraction of skeletal muscles activates specific biological pathways that significantly elevate BDNF levels in both peripheral blood and the hippocampus (Szuhany et al. 2015). Notably, during continuous aerobic contraction, a specific myokine known as Irisin is cleaved from its membrane precursor and secreted directly into the systemic circulation (Boström et al. 2012; Wrann et al. 2013). Irisin is capable of permeating the blood‐brain barrier (BBB) to directly stimulate the central nervous system to synthesize substantial amounts of BDNF (Wrann et al. 2013). This “muscle‐brain crosstalk” demonstrates that exercise directly improves hippocampal function through specific biochemical pathways, thereby enhancing learning and memory.

Alongside Irisin, Fibroblast Growth Factor 21 (FGF21) has emerged as another critical circulating factor potentially mediating this aerobic‐induced crosstalk (Pedersen 2019; Bookout et al. 2013). Upon secretion into the systemic circulation, peripheral FGF21 crosses the BBB and binds to its specific receptor complex (FGFR1/β‐klotho) within the central nervous system, targeting regions such as the hypothalamus (Bookout et al. 2013), Importantly, preclinical evidence suggests that this FGF21‐mediated signaling may extend its neuroprotective effects to core cognitive regions like the hippocampus, where it potentially activates cascades that augment synaptic plasticity and dendritic spine density, restore mitochondrial function, and attenuate brain cell apoptosis (Sa‐Nguanmoo et al. 2016). Furthermore, to better capture the complexity of how these exercise‐induced factors coordinate peripheral and central communication, recent advancements in system‐wide multi‐omics profiling, integrating the transcriptome, proteome, metabolome, and lipidome, have begun to map broader molecular adaptations to exercise (Contrepois et al. 2020). Longitudinal analyses reveal an orchestrated choreography of biological processes, demonstrating that physical activity triggers thousands of dynamic molecular changes involving energy metabolism, oxidative stress, inflammation, and growth factor responses (Contrepois et al. 2020). By profiling these system‐wide omics shifts, we hypothesize that future research may eventually elucidate how global peripheral signals could modulate central neural networks. At this stage, this multi‐omics perspective provides a promising hypothetical framework for expanding our mechanistic understanding of depressive cognitive recovery from isolated single‐target pathways to a systemic molecular network. Building upon this neurochemical foundation, recent animal studies utilizing sophisticated fiber photometry and chemogenetic techniques have mapped specific underlying neurocircuitry, demonstrating that long‐term aerobic exercise activates the oxytocinergic neural circuit of the paraventricular nucleus of the hypothalamus to the nucleus accumbens (PVN‐NAc) pathway (Xia et al. 2025). In these murine models, exercise not only restores oxytocin release within the NAc but also maintains normal neuronal excitability via the oxytocin receptor‐mediated PKD1 ERK CREB signaling cascade. While these elegant preclinical findings provide a highly compelling neurobiological framework for how physical activity might mitigate anhedonia and improve cognitive flexibility, it is imperative to exercise caution when extrapolating these precise molecular pathways directly to human pathology. Ultimately, these animal‐derived mechanisms highlight promising therapeutic targets that urgently await rigorous validation in future human clinical trials involving depressed cohorts.

Second, mechanistically, the cognitive benefits of resistance training (RT) are predominantly mediated via the insulin‐like growth factor‐1(IGF‐1) pathway (Rodríguez‐Gutiérrez et al. 2023). While the pro‐angiogenic effects of muscle‐derived IGF‐1 are well‐established, angiogenesis alone is insufficient to fully elucidate the robust improvements in structural white matter integrity. Crucially, upon crossing the blood‐brain barrier, peripheral IGF‐1 exerts profound direct neurotrophic effects on the central oligodendroglial lineage (O'Kusky and Ye 2012). Specifically, IGF‐1 binds to its respective receptors on oligodendrocyte precursor cells (OPCs), potently stimulating their proliferation, survival, and terminal differentiation into mature, myelin‐producing oligodendrocytes (Zeger et al. 2007). This targeted enhancement of myelination and remyelination processes is essential for repairing the microstructural disruption of prefrontal white matter tracts frequently observed in depressed patients. By synergistically promoting both vascular plasticity and OPC‐driven myelin renewal, this IGF‐1‐mediated mechanism forms a rigorous logical closed loop, ultimately translating peripheral mechanical stimuli from resistance training into the structural restoration of prefrontal executive functions.

Furthermore, the mechanistic rationale for concurrent training (CT) highlights a profound biological synergy. Combining aerobic and resistance exercise often triggers a physiological “interference effect” in skeletal muscle adaptations, where aerobic‐induced AMP‐activated protein kinase (AMPK) activation can inhibit resistance‐induced mammalian target of rapamycin (mTOR) signaling (Coffey and Hawley 2017). However, within the central nervous system, CT primarily yields profound synergistic effects. By combining both modalities, CT simultaneously activates the aerobic‐driven BDNF‐TrkB cascade and the resistance driven IGF‐1‐Akt neurotrophic pathways (Cassilhas et al. 2012). This dual activation creates a highly synergistic neurochemical microenvironment that simultaneously counteracts neurotoxicity and facilitates structural brain plasticity across widespread neural circuits, fundamentally explaining the superior global cognitive benefits observed in CT paradigms (Hötting and Röder 2013).

Third, mind‐body exercise (MBE) ameliorates cognitive function in depression, primarily involving the recalibration of the hypothalamic‐pituitary‐adrenal (HPA) axis. Firstly, depression is frequently accompanied by HPA axis hyperactivity and elevated cortisol levels; this state of chronic stress exerts direct neurotoxicity on the hippocampus, a region rich in glucocorticoid receptors, precipitating memory decline. Through its deep‐breathing and meditative components, MBE effectively attenuates salivary cortisol levels and mitigates neuroinflammatory responses (Bower and Irwin 2016; Pascoe et al. 2017).

Concurrently, the general physical activity regulates inflammatory factors and oxidative stress. Depressed patients are frequently in a state of chronic sterile inflammation, where high levels of pro‐inflammatory cytokines (e.g., TNF‐alpha, IL‐6) disrupt the blood‐brain barrier and induce neuronal apoptosis. In non‐depressed populations, exercise exerts a profound anti‐inflammatory effect, reducing circulating pro‐inflammatory cytokines while upregulating anti‐inflammatory mediators (e.g., IL‐10) (Gleeson et al. 2011; Guimarães et al. 2024). While physical activity is well‐documented to ultimately upregulate endogenous antioxidant enzyme systems and mitigate lipid peroxidation, describing this purely as a linear protective mechanism overlooks the subtle nuances of exercise‐induced hormesis (or mitohormesis) (Radak et al. 2008). The generation of reactive oxygen species (ROS) during exercise is dynamically regulated in a strictly intensity‐dependent biphasic manner. Moderate‐intensity exercise generates physiological levels of ROS, which act as indispensable intracellular signaling molecules to activate the Nrf2/ARE pathway, thereby upregulating endogenous antioxidant defenses and fostering synaptic plasticity. However, excessively high‐intensity or exhaustive exercise loads can transiently and severely provoke an acute overproduction of systemic oxidative stress (Radak et al. 2013). This massive ROS burst overwhelms the brain's basal antioxidant capacity, causing acute lipid peroxidation in neuronal membranes and exerting detrimental effects on synaptic plasticity (e.g., impaired long‐term potentiation) before the endogenous defenses can be adequately mobilized (Di Liegro et al. 2019; Lin et al. 2012). Therefore, acknowledging this hormetic dose‐response is crucial for optimizing exercise prescriptions and ensuring that the metabolic load stimulates neuroprotection rather than transient neurotoxicity in depressed cohorts. However, translating these robust physiological adaptations to clinical depression reveals a highly complex landscape. Recent literature indicates that empirical studies examining exercise‐induced inflammatory and oxidative changes specifically within depressed cohorts have frequently yielded mixed or null findings. Crucially, rather than indicating a true absence of biological efficacy, these null results are highly likely attributable to the pervasive low methodological quality of existing clinical trials. The current literature is heavily compromised by uncontrolled confounding factors, including unmeasured variations in baseline inflammatory status, concurrent antidepressant pharmacotherapy, and heterogeneous body mass indices, all of which obscure the true exercise response. While the optimization of this immune metabolic environment remains a highly plausible physiological basis for cognitive recovery, this prominent discrepancy in the literature strongly dictates an urgent need for high‐quality, rigorously controlled, and well‐powered longitudinal studies to definitively validate these anti‐inflammatory mechanisms in patients with depressive disorder.

Furthermore, to fully reveal the impact of systemic circulation on neuroimmunity, this peripheral crosstalk model must additionally incorporate the exercise‐induced microbiota‐gut‐brain regulatory pathway. Recent biochemical evidence highlights that exercise significantly alters the composition of the gut microbiota, promoting the enrichment of beneficial bacterial strains that produce short‐chain fatty acids (SCFAs), predominantly butyric acid, propionic acid, and acetic acid (Souza et al. 2023; Mailing et al. 2019). As cutting‐edge metabolic mediators, these SCFAs readily cross the blood‐brain barrier and exert profound neuroactive effects. Specifically, butyric acid acts as a potent epigenetic regulator (e.g., histone deacetylase inhibitor), which directly downregulates pro‐inflammatory cytokine expression in microglia and upregulates BDNF transcription (Stilling et al. 2016). By fundamentally mitigating neuroinflammation and optimizing the neuroimmune microenvironment, the SCFA‐mediated gut metabolic pathway forms a crucial, albeit frequently overlooked, biological bridge linking peripheral exercise stimuli to the amelioration of cognitive deficits in depression (Souza et al. 2023).

Finally, recent advances in cell biology have extended the protective mechanisms of exercise to the coupling of subcellular organelles and specific neurotransmitter circuits. Research utilizing cognitive impairment models has confirmed that exercise training can specifically activate the cholinergic neural circuit of the medial septum‐medial habenula (MS‐MHb) (Feng et al. 2025). Crucially, the activation of this circuit further regulates Mitochondrial Quality Control (MQC) in downstream neurons. By upregulating the expression of Mitochondrial Transcription Factor A (TFAM) and Nuclear Respiratory Factor1 (NRF1), exercise repairs damaged mitochondrial ultrastructures (e.g., ameliorating the fragmentation and swelling of mitochondrial cristae), thereby restoring neuronal energy supply and synaptic transmission efficiency (Feng et al. 2025). Given that depression is frequently accompanied by deficits in cerebral energy metabolism, this cross‐level repair mechanism spanning from neural circuits to mitochondrial biogenesis offers a novel bioenergetic perspective on how exercise improves cognitive function.

While the upregulation of these transcription factors indicates robust mitochondrial biogenesis at the gene expression level, recent evidence from high‐throughput metabolomics provides a more profound biochemical elucidation of this energy network remodeling. System‐wide metabolomic profiling reveals that exercise induces a profound metabolic rewiring across multiple organs, including the brain, characterized by the significant enrichment of tricarboxylic acid (TCA) cycle intermediates and an optimized ATP/AMP ratio (Sato et al. 2022). Crucially, the depressed brain typically exhibits severe regional glucose hypometabolism and mitochondrial dysfunction (Amin et al. 2023),In this pathological context, exercise‐induced metabolomic shifts highlight the critical activation of alternative energy pathways. Specifically, exercise significantly upregulates the astrocyte‐neuron lactate shuttle (ANLS) (Matsui et al. 2017) and enhances the cerebral utilization of peripheral ketone bodies, particularly *β*‐hydroxybutyrate (BHB) (Sleiman et al. 2016). By utilizing lactate and BHB as highly efficient alternative metabolic substrates, exercise effectively bypasses the classical glucose deficits in depression, directly refueling the depleted neuronal ATP pools (Petit and Magistretti 2016). Consequently, this metabolomics‐level remodeling of the energy metabolism network provides the solid biochemical foundation necessary to sustain the high energy demands of synaptic transmission and action potential firing within the cholinergic neural circuit.

### 5.2 From Functional to Structural Alterations

Beyond biochemical markers, electroencephalography (EEG) and neuroimaging provide direct evidence of functional and structural brain alterations, elucidating how exercise ameliorates cognition by remodeling brain architecture and optimizing neural transmission pathways.

Functionally, Event‐Related Potentials (ERPs) serve as objective indices for evaluating cognitive processing speed and resource allocation. Patients with depression typically exhibit reduced P300 amplitude and prolonged latency, reflecting deficits in attentional allocation and working memory (Polich 2007; Santopetro et al. 2025). Multiple studies have demonstrated that long‐term, regular exercise can significantly shorten P300 latency and increase its amplitude in depressed patients (Alderman et al. 2016). This indicates that exercise enhances the brain's speed in categorizing stimuli and its capacity to recruit neural resources. Furthermore, resting‐state EEG research reveals that exercise can modulate frontal alpha asymmetry, alleviating the left‐frontal hypoactivation characteristic of depression (Deslandes et al. 2010). Other studies indicate that exercise enhances frontal midline theta power, which is closely associated with sustained attention and improved executive function, suggesting that exercise likely optimizes neural oscillations related to cognitive control (Tsai et al. 2019).

At the structural level, exercise‐induced brain remodeling has been extensively corroborated by high‐resolution MRI and Diffusion Tensor Imaging (DTI). In addition to the previously mentioned increase in hippocampal volume, DTI studies have found that exercise training improves the integrity of white matter tracts, particularly the uncinate fasciculus (which connects the prefrontal cortex to the limbic system) and the superior longitudinal fasciculus. This elevation in fractional anisotropy (FA) signifies a marked improvement in the efficiency of neural signal transmission (Firth et al. 2018; Sexton et al. 2016). Concurrently, high‐resolution MRI demonstrates that exercise increases cortical thickness in the DLPFC and the anterior cingulate cortex (ACC), core brain regions governing cognitive control and emotion regulation (Wei et al. 2011; Firth et al. 2020).

In the resting state, exercise has been shown to attenuate the hyperactivation of the Default Mode Network (DMN), thereby reducing rumination. While strengthening the connectivity of the Central Executive Network (CEN) (Mulders et al. 2015). During cognitive tasks (e.g., the Stroop or N‐back task), fMRI reveals that patients exhibit more efficient activation patterns in the dorsolateral prefrontal cortex (DLPFC) and parietal regions following an exercise intervention. This manifests as completing tasks of equal difficulty with reduced neural resource consumption, reflecting an overall enhancement in “neural efficiency” (Smith et al. 2013). Additionally, by addressing the core symptom of anhedonia, exercise has been shown to enhance the striatum's response to rewarding stimuli, thereby repairing the impaired reward circuit (Hanuka et al. 2023). While general physical activity robustly improves these executive and reward networks, Mind‐body exercise exerts a highly specific regulatory impact on the Default Mode Network (DMN). Studies indicate that mindful movement training enhances functional connectivity between the anterior cingulate cortex (ACC) and the insula, thereby heightening interoception (awareness of the present moment) and suppressing aberrant DMN activity. This attentional shift from “internal absorption” to “external experience” liberates working memory resources previously hijacked by ruminative thinking, allowing them to be reallocated to executive control tasks. This network‐level reallocation likely represents the core neural mechanism through which MBE enhances attention and decision‐making capabilities in depressed patients.

### 5.3 Psychological and Behavioral Pathways: The Mediating Roles of Self‐Efficacy and Emotion Regulation

Primarily, the amelioration of mood and the elevation of self‐efficacy serve as crucial catalysts for cognitive recovery. Exercise training not only ameliorates the affective state of patients with depression but also fosters the restoration of cognitive function by augmenting self‐efficacy (Hird et al. 2024). Research indicates that depressed patients frequently experience profound low mood and diminished self‐efficacy, both of which exert detrimental effects on cognitive function (Zhou et al. 2025). Through exercise training, patients experience enhanced physical capabilities and positive affective shifts, thereby bolstering their self‐efficacy and augmenting their confidence and sense of mastery over activities of daily living (Hird et al. 2024). A significant positive correlation exists between this heightened self‐efficacy and cognitive recovery. For instance, one study found that older adults with depression who engaged in aerobic exercise training exhibited significant post‐intervention cognitive improvements, particularly in executive function and memory; crucially, this enhancement was intimately correlated with the amelioration of their affective state (Song and Yu 2019). Studies further suggest that cognitive impairment in depression may be linked to functional aberrations in the prefrontal cortex and the hippocampus regions inextricably involved in both emotion regulation and cognitive processing (Masse et al. 2022). Exercise training potentially ameliorates the functionality of these specific brain regions, thereby facilitating cognitive recovery. Moreover, exercise may underpin cognitive enhancement by improving vascular health and increasing cerebral blood flow (Pereira et al. 2007; Davenport et al. 2012).

Furthermore, the social interaction and psychological support derived from exercise synergistically augment the efficacy of cognitive training. Exercise not only furnishes depressed patients with abundant opportunities for social engagement and psychological support (Stevens et al. 2021) but also plays a pivotal role in potentiating the outcomes of cognitive rehabilitation. Evidence demonstrates that social interaction effectively mitigates depressive symptoms (Dingle et al. 2021), and facilitates cognitive recovery by elevating mood and fostering a sense of belonging. Depressed patients participating in group sports or collective training not only receive support and encouragement from peers but also forge social connections and strengthen their social support networks through shared physical experiences (Jetten et al. 2022). In an exercise intervention study targeting depressed patients, participants engaging in group aerobic exercise universally reported superior mood amelioration and cognitive enhancement, with improvements in attention and processing speed being particularly pronounced (Alizadeh Pahlavani 2024; Haussleiter et al. 2020). Additionally, the confluence of positive psychological effects and social interaction engendered by exercise helps establish a virtuous feedback loop, propelling patients toward dual progress in both affective and cognitive domains (Schmitter et al. 2025) Consequently, integrating the social dimensions of exercise not only elevates the patient's physical experience but also cultivates a highly conducive environment for cognitive recovery, a paradigm that warrants further promotion and exploration in clinical practice (Wunram et al. 2021).

### 5.4 Integrated Mechanistic Model: A Testable Interactive Regulatory Loop

Based on the synthesis of existing evidence, this study proposes a falsifiable cross‐scale “neuro‐bio‐psychological” hypothetical mechanistic model. Moving beyond static evidence chaining, this model explicitly defines the core nodes and time windows of intervention effects, and develops specific pathway hypotheses for key predictors and mediating variables (Figure 1).

**FIGURE 1:**
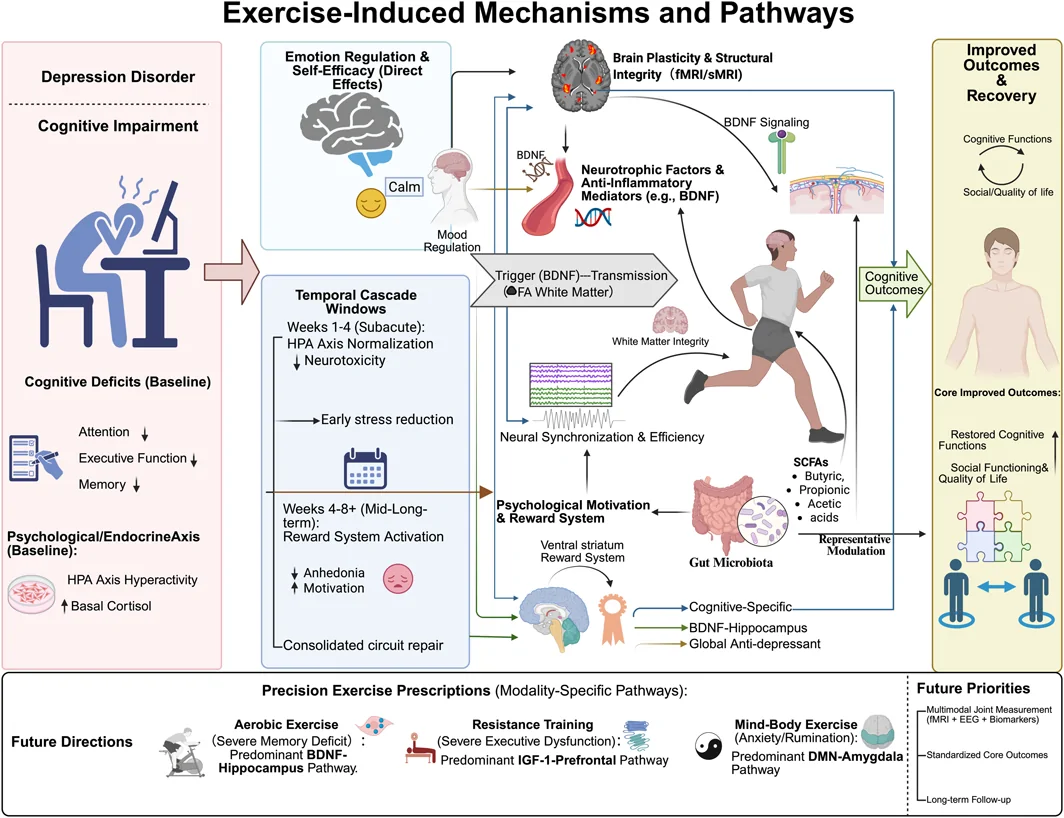
“Neuro‐bio‐psychological” hypothetical mechanistic model.

#### 5.4.1 Core Nodes and the “Molecule‐Structure‐Function” Mediation Hypothesis

Exercise‐induced cognitive improvements are not isolated, parallel events across different biological levels; rather, they exhibit significant cross‐level “crosstalk” effects. The potential core causal chain can be described as follows: “neurotrophic factors drive myelination→enhanced white matter integrity→optimized neural conduction efficiency.” By extracting three measurable key nodes, we propose the following mediation hypotheses.

1. Nodes and Transformation Pathways: Trigger layer (increased peripheral BDNF)→ Transmission layer (elevated fractional anisotropy [FA] of white matter via DTI scans)→ Output layer (optimized EEG characteristics and behavioral improvements).
2. Structural Mediation Hypothesis (Molecule‐to‐Structure): This model predicts that an isolated increase in peripheral blood BDNF is insufficient to directly explain cognitive improvement, particularly given the ongoing academic debate regarding whether systemic BDNF can cross the blood‐brain barrier (BBB) at physiologically relevant concentrations (Pan et al. 1998). Instead, current neurobiological consensus posits that exercise load drives cognitive recovery by stimulating the de novo synthesis of BDNF directly within the central nervous system (Pedersen 2019). Specifically, BBB‐permeable peripheral factors released during muscle contraction, such as Irisin, Cathepsin B (Moon et al. 2016), and energy metabolites efficiently penetrate the brain and act on central neural networks to locally upregulate hippocampal and cortical BDNF expression (El Hayek et al. 2019). Once synthesized locally, this central BDNF activates the TrkB receptor signaling pathway to regulate the proliferation of oligodendrocyte precursor cells (OPCs) and accelerate central nervous system remyelination (Fletcher et al. 2018; Predovan et al. 2021). Therefore, the change in FA (ΔFA) within critical anatomical tracts, such as the uncinate fasciculus, serves as the core structural mediator linking ΔBDNF and ΔCognitive Function (Oberlin et al. 2016).
3. Electrophysiological Mediation Hypothesis (Structure‐to‐Function): The integrity of the white matter microstructure provides the physical foundation for the efficient transmission of electrophysiological signals. This model predicts that the enhancement of cerebral information processing speed is directly mediated by a “shortened P300 latency,” whose physical prerequisite is the increase in FA (Matsushita et al. 2011; Fjell et al. 2011). Strengthened white matter fibers reduce signal attenuation and improve the signal‐to‐noise ratio (SNR) during long‐distance transmission, enabling the recruitment of more neural resources during task execution (manifested as increased P300 amplitude) (Yang et al. 2020; Li et al. 2020).

Therefore, the multimodal mechanisms by which exercise ameliorates cognitive function in depression can be epitomized as follows: exercise‐induced BDNF signaling, by promoting oligodendrocyte‐mediated myelination (structural repair), physically augments the conduction velocity and synchronization of neural circuits (functional optimization), ultimately culminating in the behavioral amelioration of executive function and memory.

#### 5.4.2 Expected Time Windows: The Temporal Cascade Effect of Neuro‐Psychological Interactive Regulation

Beyond unidirectional biological pathways, the psychological and neuroendocrine systems form a dynamic feedback loop. It is important to note that the chronological sequence detailed below represents a synthesized theoretical framework derived from current cross‐sectional and pre‐post intervention evidence, rather than a continuously tracked trajectory within a single cohort. Although substantial empirical data independently corroborate the rapid onset of endocrine shifts (Beserra et al. 2018) and the delayed manifestation of structural and functional neural network remodeling (Brush et al. 2022), comprehensive longitudinal trials employing synchronous multimodal measurements (e.g., continuous biomarker tracking coupled with sequential fMRI scans) are still required to fully validate this temporal cascade in a single cohort. Therefore, this timeline serves as a highly testable predictive model for future precision exercise research.

1. Acute/Subacute Phase (Weeks 1–4 of Intervention): HPA Axis Normalization. The psychological stress‐reducing effect during the early stages of exercise rapidly feeds back into the endocrine system. It is expected that a reduction in hypothalamic‐pituitary‐adrenal (HPA) axis hyperactivity and a decrease in basal cortisol levels will be observed first (Budde et al. 2010). This early hormonal regulation mitigates neurotoxicity, providing a protective window for subsequent hippocampal neurogenesis.
2. Mid‐to‐Long‐Term Consolidation Phase (Weeks 4–8+ of Intervention): Structural Activation of the Reward System. As the sustained effects of long‐term exercise are established, a significantly enhanced striatum to reward anticipation is expected to be observed via fMRI (Basso and Suzuki 2017). This propelling mid‐to long‐term circuit repair, driven by psychological states, not only ameliorates anhedonia but also serves as a crucial motivational mediator, directly promoting the recovery of executive functions.

#### 5.4.3 Modality Specificity and Baseline Predictors

Based on the aforementioned closed‐loop model, this model explicitly posits that different exercise modalities operate through distinct multimodal pathways. This framework provides testable baseline predictors for future precision exercise prescriptions.

1. Patients presenting with severe impairments in long‐term and spatial memory at baseline exhibit a robust therapeutic response to aerobic exercise. This modality distinctively maximizes BDNF release to drive hippocampal volume expansion (Voss et al. 2013); however, its neuroplastic effects operate synergistically across large‐scale brain networks rather than in anatomical isolation. Extensive neuroimaging evidence demonstrates that aerobic exercise concurrently increases cortical thickness in the dorsolateral prefrontal cortex (DLPFC) and enhances the functional connectivity of the central executive network (Colcombe et al. 2006). Therefore, while the “BDNF‐Hippocampus” axis represents the predominant biological signature of aerobic training, this modality simultaneously confers significant global benefits for executive control.
2. Predictive Pathway for Resistance Training: Conversely, patients exhibiting severe executive dysfunction and attention deficits at baseline show highly optimized responses to resistance training. Muscle contractions trigger the specific release of IGF‐1, which traverses the blood‐brain barrier to promote angiogenesis and targeted prefrontal remyelination, directly correlating with improved white matter integrity (Cassilhas et al. 2007). Crucially, this IGF‐1‐prefrontal pathway operates in tandem with the aforementioned cortical adaptations. This modality‐specific framework acknowledges a vital clinical reality: while aerobic and resistance exercises possess distinct neurobiological emphases, their structural remodeling effects dynamically overlap to collectively rescue the depressed connectome (Schuch et al. 2016; Voss et al. 2010; Batouli and Saba 2017).
3. Predictive Pathway for Mind‐Body Exercise: For patients whose baseline is characterized by severe anxiety, excessive rumination, and HPA axis hyperactivity, mind‐body exercises (e.g., Tai Chi, Yoga) represent the optimal predictive intervention modality. By emphasizing the coordination of breath and attention, these modalities can attenuate abnormal connectivity between the default mode network (DMN) and the emotional center (the amygdala), while increasing insular thickness, thereby prioritizing the restoration of emotion regulation and interoceptive awareness (Tao et al. 2017).
4. Predictive Pathway for Concurrent Training (CT): For patients presenting with pervasive cognitive deficits spanning both memory and executive domains at baseline, concurrent training represents the most comprehensive intervention. While classic exercise physiology often warns of a peripheral “interference effect” in skeletal muscle adaptations (where aerobic‐induced AMPK activation inhibits resistance‐induced mTOR signaling) (Coffey and Hawley 2017), this paradigm shifts entirely within the central nervous system. By systematically combining modalities, CT primarily yields profound synergistic effects, simultaneously activating the aerobic‐driven BDNF‐TrkB cascade and the resistance‐driven IGF‐1‐Akt neurotrophic pathways (Cassilhas et al. 2012). This dual activation creates a highly synergistic neurochemical microenvironment that facilitates structural brain plasticity across widespread neural circuits, fundamentally overriding peripheral interference (Hötting and Röder 2013).

In summary, exercise does not act through a single target but rather through the integration of peripheral regulation by blood biochemical factors, central remodeling of brain functional networks, and the mediating regulation of psychological states. Together, they form a complex “neural‐psychological‐behavioral” network to collectively reverse depression‐related cognitive impairments. This multimodal mechanistic framework not only elucidates the efficacy of exercise interventions but also provides a theoretical basis for developing personalized exercise prescriptions based on biomarkers and neuroimaging in the future.

## 6 Individual Heterogeneity and Precision “Targeted” Interventions

The therapeutic efficacy of exercise interventions exhibits pronounced heterogeneity across diverse populations of patients with depression, heavily modulated by individual demographic and clinical differences. To maximize therapeutic outcomes, the formulation of future exercise prescriptions must transcend traditional “mean‐based strategies.” A recent Bayesian model‐based network meta‐analysis evaluating optimal exercise doses (Ge et al. 2025) explicitly highlights the necessity of accounting for demographic heterogeneity. Recognizing these moderating variables, especially how factors like age and sex alter treatment efficacy, is a clinical imperative for designing truly individualized, precision exercise prescriptions that explicitly highlight the clinical imperative of accounting for demographic heterogeneity. Recognizing these moderating variables enables the construction of multidimensional precision intervention protocols. Specifically, this paradigm shift entails addressing the following core dimensions.

1. Age‐Related Disparities: Significant disparities exist between younger and older patients regarding physiological and psychological responses. Younger patients may recover cognitive function more rapidly following exercise, whereas older adults often require extended periods and adaptive adjustments to achieve comparable benefits (Yan and Li 2025; Toval et al. 2025). Interestingly, studies show that when engaging in moderate‐intensity aerobic exercise, older depressed patients frequently experience particularly pronounced cognitive enhancements compared to their younger counterparts, underscoring the absolute necessity of age‐stratified protocols (Gökçe et al. 2019; Monteiro et al. 2021; Dillon et al. 2022; Dhahbi et al. 2025). (2) Sex‐Specific Responses: Biological sex distinctly interacts with exercise intensity, cumulative volume, and neuroendocrine adaptations. Neurobiologically, variations in gonadal hormones (e.g., fluctuations in estrogen and testosterone) can significantly modulate exercise‐induced neurogenesis and BDNF expression (Szuhany et al. 2015; Barha et al. 2017). Furthermore, males and females often exhibit differential physiological adaptations (Ansdell et al. 2020; Weber et al. 2024). Consequently, implementing sex‐tailored prescription parameters is essential to optimize both central neuroplasticity and therapeutic compliance. (3) Symptom Severity: The severity of depression essentially modulates exercise efficacy. Studies reveal that patients with severe depressive symptoms, despite overall improvements, exhibit substantially lower magnitudes of cognitive enhancement compared with those with mild depression. This indicates that symptom severity is an indispensable factor in personalized exercise interventions, necessitating lower‐intensity “gradual progression” strategies to prevent excessive physiological loads that could exacerbate psychological frustration (Guan et al. 2024; Wu et al. 2025). (4) Depression Subtypes: Tailored interventions grounded in neurobiological mechanisms must be implemented for heterogeneous depression subtypes: Anxious Distress: Characterized by HPA axis hyperactivation (Vinkers et al. 2021; Fiksdal et al. 2019). These patients require a “calming strategy.” Mind‐body exercises (e.g., yoga, Tai Chi) or moderate‐intensity aerobic exercise should be prioritized (Xu et al. 2025) to activate the parasympathetic nervous system and downregulate cortisol levels. Caution is warranted regarding high‐intensity anaerobic training, which may precipitate somatic anxiety responses (Li et al. 2019). Psychomotor Retardation: Characterized by dopaminergic hypofunction and avolition (Felger and Treadway 2017; Buyukdura et al. 2011). These patients benefit from an “activation strategy.” Therefore, resistance training, complex motor skill exercises, and high‐intensity interval training (HIIT) should be prioritized. While resistance training stimulates the reward circuit via muscle contraction and “forced use” mechanisms (Toval et al. 2025), HIIT serves as a potent physiological stressor that acutely triggers a robust release of the catecholamine system, thereby rapidly activating the blunted dopaminergic pathways (Kliszczewicz et al. 2018; Zouhal et al. 2008; Ghanbari et al. 2022). By synergistically employing these modalities, this activation strategy can effectively ameliorate anhedonia, improve motor efficiency, and restore executive function, thereby improving motor efficiency and executive function (Hird et al. 2024). (5) Typology of Cognitive Impairment: The specific nature of the cognitive deficit critically influences intervention outcomes. For example, patients with mild cognitive impairment manifest significant cognitive amelioration following regular aerobic exercise. Conversely, those with severe comorbid conditions (e.g., Alzheimer's disease) may exhibit blunted cognitive responses to exercise or even negligible improvements in certain instances. Such cases frequently necessitate the integration of cognitive training (e.g., dual‐task training) to overcome therapeutic bottlenecks (Dell'Osso et al. 2024; Liu et al. 2025). (6) Lifestyle Factors: Variables such as prior exercise habits, dietary patterns, and social support further impact intervention efficacy. Research indicates that individuals with robust social support typically achieve more pronounced cognitive interventions (Stuckenschneider et al. 2021; Gaudreau‐Majeau et al. 2025).

Therefore, to achieve optimal exercise intervention outcomes, prescription protocols must transcend static templates and serve as a dynamically adjustable closed‐loop system. Real‐time monitoring and fine‐tuning based on the patient's physiological biomarkers and behavioral manifestations are imperative. This precision model of “assess‐intervene‐reassess” not only significantly bolsters intervention effectiveness but also robustly mitigates exercise‐associated risks, ensuring that every patient reaps maximal cognitive benefits within their optimal physiological and psychological window.

## 7 Future Research Directions and Multidisciplinary Cross‐Application

Moving forward, the mechanisms by which exercise training improves cognitive function in patients with depression warrant deeper and more rigorous investigation. To advance the field from empirical observation to precision medicine, future research should prioritize the following multidimensional trajectories: First, the combined evaluation of exercise efficacy utilizing multimodal neuroimaging and biological markers will provide a critical foundation for understanding the underlying mechanisms. As synthesized in this review, exercise interventions mitigate cognitive deficits through diverse biological pathways. Future research must integrate micro‐level (e.g., peripheral blood biomarkers) and macro‐level (e.g., structural and functional brain networks) assessments. This comprehensive approach will clearly elucidate the biological basis of exercise interventions, thereby providing robust scientific support for the development of highly personalized exercise prescriptions. Second, continuous monitoring of biomarkers will help elucidate the neurobiological trajectory of exercise and its preventative potential against long‐term cognitive decline. Importantly, a multidisciplinary collaborative model that strategically combines psychotherapy, pharmacotherapy, and exercise training has been proven to significantly optimize therapeutic outcomes.

In summary, future paradigms should focus on the application of multimodal assessment technologies, the innovation of digital health interventions, and the execution of longitudinal tracking studies. Concurrently, the comprehensive management of depression mandates a multidisciplinary synergy that extends beyond clinical treatments to encompass robust community and family support. Exploring how to effectively integrate these diverse therapeutic modalities to deliver optimized, individualized care remains a crucial frontier for future research.

## 8 Conclusion

As a non‐pharmacological behavioral intervention, exercise training has demonstrated stable and clinically significant potential in ameliorating cognitive function in patients with depression. The beneficial effects of exercise do not originate from a single pathway but rather arise from a “multi‐level and multi‐system” synergistic mechanism. However, it must be emphasized that significant heterogeneity remains across different studies regarding sample characteristics, exercise prescriptions, outcome measures, and follow‐up durations. Furthermore, the causal chains and temporal sequences among multimodal indices have yet to be fully elucidated.

Future research should, predicated on standardized assessment frameworks, conduct higher‐quality randomized controlled trials (RCTs) and long‐term follow‐ups, while propelling the joint analysis of multimodal data: On the one hand, through the synchronous acquisition and dynamic tracking of neuroimaging, peripheral biomarkers, and electrophysiological signals, studies must clarify which indicators best predict cognitive benefits and which alterations serve as key mediators. This will enable a deeper exploration of the dose‐response relationship between exercise training, depression, and cognitive function. On the other hand, integrating digital health and wearable technologies can enhance the accessibility, adherence, and sustainability of exercise interventions in real‐world settings.

Ultimately, by combining objective physiological biomarkers with clinical phenotypes to establish interpretable and verifiable stratified models, exercise therapy can transition from “empirical recommendations” to “quantifiable, predictable, and individualized” precision prescriptions. This paradigm shift will provide a highly operational evidence‐based foundation for the cognitive rehabilitation of patients with depression.

## Author Contributions

Z.G., F.B.S., and X.W. methodology: Z.G., F.B.S., X.W., J.J.X.L., Y.D., and L.D. investigation: W.L., W.J., C.L., S.J., Y.S., F.D., Z.X., M.Y., K.Z., X.M., P.W., and C.W. visualization: Z.X. and Z.G. writing–original draft: Z.G., L.D., J.J.X.L., Y.D., and F.B.S. writing–review and editing: F.B.S. and X.W. supervision: F.B.S. and X.W. project administration: X.W. and L.D. All authors have read and agreed to the published version of the manuscript. Z.G., L.D., J.J.X.L., and Y.D. contributed equally to this work.

## Funding

This work was supported by Shanghai Baoshan District Health Commission Excellent Youth (Yucai) Program (BSWSYC ‐2023 ‐19).

## Ethics Statement

The authors have nothing to report.

## Consent

All authors consented to the publication of the manuscript.

## Conflicts of Interest

The authors declare no conflicts of interest.

## Data Availability

Data sharing not applicable to this article as no datasets were generated or analyzed during the current study.
